# Reflections on a career as an amateur crystallographic software developer

**DOI:** 10.1063/4.0001233

**Published:** 2026-11-03

**Authors:** Brian H. Toby

**Affiliations:** Advanced Photon Source, Argonne National Lab, 9700 S. Cass Ave., Lemont, Illinois 60439, USA

## Abstract

The author recaps software development over the course of his career and how George Sheldrick inspired and improved this. He concludes that software development is more accepted at present as a role for research scientists, but that more consideration is needed for long-scale project development.

Like many of the people reading this, I was introduced to crystallographic computing in large part relying on software from George Sheldrick. Unlike many of you, I was using computer cards. It is hard to imagine those days now. Back then, one might wait for hours before the computer would start to run a larger (say, a half-megabyte) computer program. The entire memory of the university's single IBM 360 computer was then on the order of a megabyte or two, and the computer took up most of the building's basement. Many programs would be loaded into memory at the same time, so that while one program was waiting for access to information on disk or tape, another program could be run. The programs that would hog too much memory were usually delayed for overnight runs. Output was obtained on wide folded-paper printouts. The computers were slow, but the printers were fast. A 20-kilo box of paper could be consumed in a fraction of an hour. Sigh for the trees. The first page of the printout included a bill for the time and resources it took your program to run. The computer budget could not be spent on anything else, so we called it “funny money,” but the charge for a single hour of computer time was probably close to a month of my undergraduate summer salary. Back then, scientists spent days and sometimes weeks optimizing programs to run faster and consume less memory. George was a master at those improvements. One skill from those days that I will never use again was learning to create “overlay structures” that designated different parts of programs that could share memory, thus reducing requirements. Yes, much has changed.

What has not changed is that innovative software leads science in new directions and allows us to obtain improved results and sometimes even previously impossible results. AI can now help us generate code and perhaps will someday preempt us mortal and oh-so-limited humans from the job of coding, but I do not think AI will remove humans from innovation and technique optimization.

What has changed, at least from my perspective, is the role that software development has in career growth for scientists. As an undergrad, I was already active in developing single-crystal software. In addition to my work in the crystallography group, I contributed software to three other labs as an undergraduate. I can remember two of those faculty members telling me how profoundly my work automating instruments improved their research. (The third, my father, never did, but his other benefits to my life are myriad.) Despite these significant accomplishments, I was rightfully discouraged from highlighting them alongside my undergrad co-authorships when applying for grad school. Computation, in terms of both hardware and software development, was seen as a craft and not proper science. The fear was that if people saw that I did scientific software development, then they would not perceive me as an active research scientist. That, I believe, has changed for the better.

In my grad school days, software was something that I did to further my own lab work or for side income. Programming work for Exxon paid for my family visits to New Jersey from California. Alas, back then I did not appreciate the level of accomplishment achieved by the surface-science modeling code that I wrote for my Ph.D. research. The goal was to model some rather unusual $N_{2}$ adsorption and desorption data from the Ru(0001) surface. The model was a cartoon—the physics, based on ideas from a postdoc collaborator, was overly simplistic—but the computations reproduced every unexpected and previously unexplained result we had obtained. To me, back then, it was just some software. Alas, I believe, my advisor thought so too. Now, with more experience, I understand how uncommon it is for a model to reproduce data with such fidelity, and explain so much. The lack of recognition for this almost cost me a completed thesis and degree. This was no small matter.

After grad school, I started a dead-end job as a staff scientist at Union Carbide Corp., but that did start my transition to powder diffraction. Carbide sent me to Brookhaven National Lab to learn more about powder diffraction, which changed my life so much for the better. I had the very great fortune there to learn from three wonderful scientists, and, for that matter, wonderful human beings, David Cox, Larry Finger, and Takeshi Egami. Thanks to Dave, I had the chance to learn a bit about neutron diffraction and a lot about synchrotron diffraction—as it was being invented. Also from Dave, I was able to learn about Rietveld refinement. He valued my programming skills, both at the beamline and in his Rietveld code, and I got started with zeolite crystallography with him at BNL, something somewhat ironic considering that Union Carbide pretty much invented commercial zeolite development. The sum of those areas pretty much launched my future science career. I later worked for Takeshi; another high point in my life.

Larry Finger served as a role model of a truly fine scientist who also had incredible computer skills. Larry's science was cutting-edge and impeccably done, but he also created superb software. He was also masterful with computational hardware. He automated the first-ever dedicated high-resolution synchrotron powder diffractometer (NSLS's X13A) by building a PDP-11 computer basically from spare parts, as the budget did not have the $50 K–$100 K needed for a new computer. I did have the chance to tell Larry how important he was to me as someone I wanted to emulate. He was honored but also taken aback, as I think he saw me as a peer. Larry was quite humble.

While at Union Carbide, I made the decision that since doing publishable research there was impossible, I could “publish” software, so I wrote a package of VAX programs for powder diffraction processing called the Powder Suite (knowing that this term was a British euphemism). It was popular enough that one instrument manufacturer, without permission, packaged it with their hardware. I believe that the Powder Suite pioneered the idea of using multiple logical criteria as a way to locate entries in the ICDD database. I think this is now called the PDF-4 technique. Much of the Powder Suite was useful enough that I re-coded much of it a decade or so later in a Tcl/Tk GUI, as a program called CMPR, which had many fans in its day.

From Union Carbide, my next stop was as a postdoc and eventually a lecturer at the University of Pennsylvania. Yes, most people go to industry after a postdoc, not before, but my life has not exactly been conventional. My plan had been to work on quasicrystals with Takeshi, but the newly discovered high-Tc superconductors hijacked that. I ended up working on local structure using PDF techniques. I cannot claim that I was involved in inventing PDF work, as the groundwork for that was done long before I was born, but I did write the first PDF-fitting program for looking at how local structure could differ from the long-range crystallographic structure.

From U of P, I went back to industry for a much more enjoyable position, this time at Air Products, where I was hired to work in powder diffraction crystallography. I got to do really exciting science, which, alas, I could not then talk about. APCI pushed me out of my VAX comfort zone by encouraging me to learn molecular modeling and purchasing for my use a Silicon Graphics Unix workstation, which cost probably ten times what my car was worth; the site license for the Biosym software cost much more. To fit both the SGI and my microVAX into my office, I had to put the CPUs into the breakroom next door and run cables through the wall. I am told that APCI is no longer the same, but it was a great place to do science and even some programming.

Then I moved to NIST, where I worked with Judy Stalick to support users at the workhorse BT-1 neutron diffractometer, and there I started my own independent research career. My software experience did me well during my decade at NIST, as computerization was key to keeping visiting users happy and productive. I then moved from NIST to Argonne to launch the 11-BM high-resolution powder diffractometer. Based on my NIST and BNL experience, I knew that software would be key to the success or failure of that project. I wrote the mail-in system myself, as management told me that we would never have the resources to have the software professionals create it, but without good software, the demands would have been too great to offer mail-in as an option. Two decades later, 11-BM is still the highest resolution and most productive powder diffraction instrument in the country, among the most productive synchrotron instruments in the world, and the mail-in access software has been cloned to its neighboring diffraction instruments.

With my move to Argonne, I also became a scientific manager and discovered that work from that job was endless, but data analysis software development was something that I could do in my spare time. So, what time I did have went into creating GSAS-II, a comprehensive crystallographic single-crystal and powder diffraction data reduction and analysis package, alongside my work-partner Bob Von Dreele. I have now escaped from management, but GSAS-II can still consume all the time I have available, and then some.

Despite my career goal of being seen as an experimentalist and researcher who uses x-ray diffraction to study materials, scientific software has come to dominate my life. I have received more than my share of awards in science, two alone in 2026, but ironically, considering my attempts to keep my software development work behind the curtain, all the awards I have gotten at least in part recognize my contributions to software. Also ironic, my “greatest hit” paper, with nearly 9000 total citations, and one that still receives >400 citations/year, is for software that I now feel is obsolete and where I discourage its use.

To bring this discussion back to George Sheldrick, I would like to return to thoughts on single-crystal diffraction. There is no doubt that single-crystal work has been revolutionized in the past decade or two, with changes that I believe have been as profound as the monumental transformation that took place in the first 50 years of crystallography. Instrumental improvements have been one major part of the recent advances, but software that offers results in minutes, and the very significant improvements in the diffraction models to offer better fitting of diffraction intensities, were also crucial to this revolution. George Sheldrick is not solely responsible for each of those, but I think he accrues more credit than anyone else.

George was willing to take on work that he saw as needing to be done, and he did not look back over his shoulder to see what recognition he would get. He did it very well. His work demonstrated that software development is a valuable and creative part of scientific advancement: It takes imagination to look at data and scientific ideas and come up with a process that enables scientists to do something useful, perhaps even something previously impossible. Even cynics need to acknowledge that the importance of software is demonstrated through citations. Fortunately, it is also, at least for some of us, fulfilling and sometimes just fun. Scientific software no longer needs to be hidden as a hobby, and for this I very much thank George, who was truly a trailblazer and an inspiration.

Looking forward, I see good things and not so good things. Scientists are now receiving career recognition for their contributions to science through software development. Software can now be an important cornerstone for building a research career. This is good for scientists and good for the advancement of science. What concerns me is that it seems that every software paper is for a new “package.” New ideas are important. One of my goals in GSAS-II, achieved successfully, was that we would provide a test bed where scientists could experiment with their novel concepts without having to first duplicate all the “messy” (calculational) bits. However, major projects require a sustained effort, and it can take considerable time before a code can be improved to meet the needs of its scientific users. This will not happen if every scientific software package is a new one.

What seems to me as missing is support for the long-term software development life cycle. While not every project will have the longevity or impact of the various incarnations of George's SHELX, in a modern environment, major projects need sustained institutional commitment. The expectations for software projects have only increased. Users want scientific software to have the same features and be as easy to use as projects produced by large commercial teams for general use. Fortunately, Python has really improved the productivity and scope of things that even an amateur crystallographic software developer, such as I deem myself, can do. However, in truth, software packaging, distribution, graphical user interface development, visualization, and so on, are specialty tasks. For productivity and quality, development of major packages is best done by teams that include software engineers working alongside scientists. Teams must be large enough to anticipate the inevitable turnover of personnel without too much loss in institutional memory. The concept of committing to the long-term development of scientific software projects and provisioning them for effective development seems not to be among the goals of scientific planning, at least not in the country where I live and work. I hope this will also change.

## Data Availability

Data sharing is not applicable to this article as no new data were created or analyzed for this report.

